# Exploring the Parkinson patients’ perspective on home-based video recording for movement analysis: a qualitative study

**DOI:** 10.1186/s12883-019-1301-y

**Published:** 2019-04-27

**Authors:** N. M. de Vries, K. Smilowska, J. Hummelink, B. Abramiuc, M. M. van Gilst, B. R. Bloem, P. H. N. de With, S. Overeem

**Affiliations:** 1 Department of Neurology, Radboud university medical center, Donders Institute for Brain, Cognition and Behaviour, P.O. Box 9101, 6500 HB Nijmegen, the Netherlands; 2 Eindhoven University of Technology, Electrical Engineering, Eindhoven, the Netherlands; 3 Eindhoven University of Technology, Sleep Medicine Centre Kempenhaeghe, Heeze, the Netherlands

**Keywords:** Parkinson’s disease, Video, Monitoring, Acceptability

## Abstract

**Background:**

Parkinson’s disease is a complex neurological disorder characterized by a variety of motor- as well as non-motor symptoms. Video-based technology (using continuous home monitoring) may bridge the gap between the fragmented in-clinic observations and the need for a comprehensive understanding of the progression and fluctuation of disease symptoms. However, continuous monitoring can be intrusive, raising questions about feasibility as well as potential privacy violation.

**Methods:**

We used a grounded theory approach in which we performed semi-structured interviews to explore the opinion of Parkinson’s patients on home-based video recording used for vision-based movement analysis.

**Results:**

Saturation was reached after sixteen interviews. Three first–level themes were identified that specify the conditions required to perform continuous video monitoring: Camera recording (e.g. being able to turn off the camera), privacy protection (e.g. patient’s behaviour, patient’s consent, camera location) and perceived motivation (e.g. contributing to science or clinical practice).

**Conclusion:**

Our findings show that Parkinson patients’ perception of continuous, home-based video recording is positive, when a number of requirements are taken into account. This knowledge will enable us to start using this technology in future research and clinical practice in order to better understand the disease and to objectify outcomes in the patients’ own homes.

## Background

Over 100 years ago, at the turn of the twentieth century, Gheorghe Marinescu video recorded patients with various neurological conditions, including Parkinson’s disease (PD) in the garden of Pantelimon Hospital, mostly for educational purposes [1–4]. While this was unique at the time, nowadays, video recording is widely used as a diagnostic, therapeutic and also monitoring tool in the clinical or research environment. In movement disorders clinics, patients are frequently asked to be video recorded during the neurological examination. This allows neurologists to consult with other specialists on specific signs or symptoms (for example, to confirm the diagnosis) or to explore other diagnostic options without requiring the patient’s presence. Moreover, videos could contribute to a better understanding of movement patterns and have, thereby, great potential as a research tool.

While still the mainstay assessment ‘tool’, neurological examination and observation in clinical settings may not always reflect the complexity and actual impact of the disease. Symptoms may for example be influenced by stress related to the consultation (e.g. leading to increased tremor, or conversely, to suppression of freezing of gait) or fluctuate during the day [5]. The severity and impact of motor symptoms are often quantified using the Unified Parkinson’s Disease Rating Scale (UPDRS) – III (motor subscale). However, the UPDRS-III is based on the subjective rating of the observer. More objective mobility measures give additional information [6–9]. Examples of objective clinical tests include the Timed Up & Go Test, the Pegboard Dexterity Test and Finger Tapping Test [10, 11]. In addition, an increasing number of spatiotemporal and kinematic parameters of gait and postural control (e.g. gait speed, stride length, cadence, joint angles, arm swing and trunk rotation) are being identified that can reliably be assessed in a laboratory setting using advanced registration systems (such as the VICON system) or body worn sensors such as accelerometers [12]. However, objective and longitudinal assessment of mobility-related parameters in the patient’s home environment is still very difficult at the moment.

Smartphones with integrated cameras allow patients to capture home videos of paroxysmal or fluctuating symptoms. In daily clinical practice, patients often share their self-made recordings with their physician to better characterize the symptoms as they present and fluctuate at home. Obviously, not only the paroxysmal or fluctuating symptoms are interesting to physicians, recordings of normal daily behaviour may provide a more realistic and accurate idea of patient functioning in daily life.

Continuous video-based technology used in the home situation may bridge the gap between the limited and fragmented in-clinic observations on the one hand, and the need for a comprehensive understanding of the progression and fluctuation of disease symptoms on the other. However, continuous video monitoring at home raises ethical concerns about privacy of patients and data security of the recordings. In order to gain design requirements for the proposed system, we studied the barriers and facilitators as perceived by PD patients considering continuous video recording at home for medical research and/or medical treatment purposes.

## Methods

### Design and participants

**Fig. 1:**
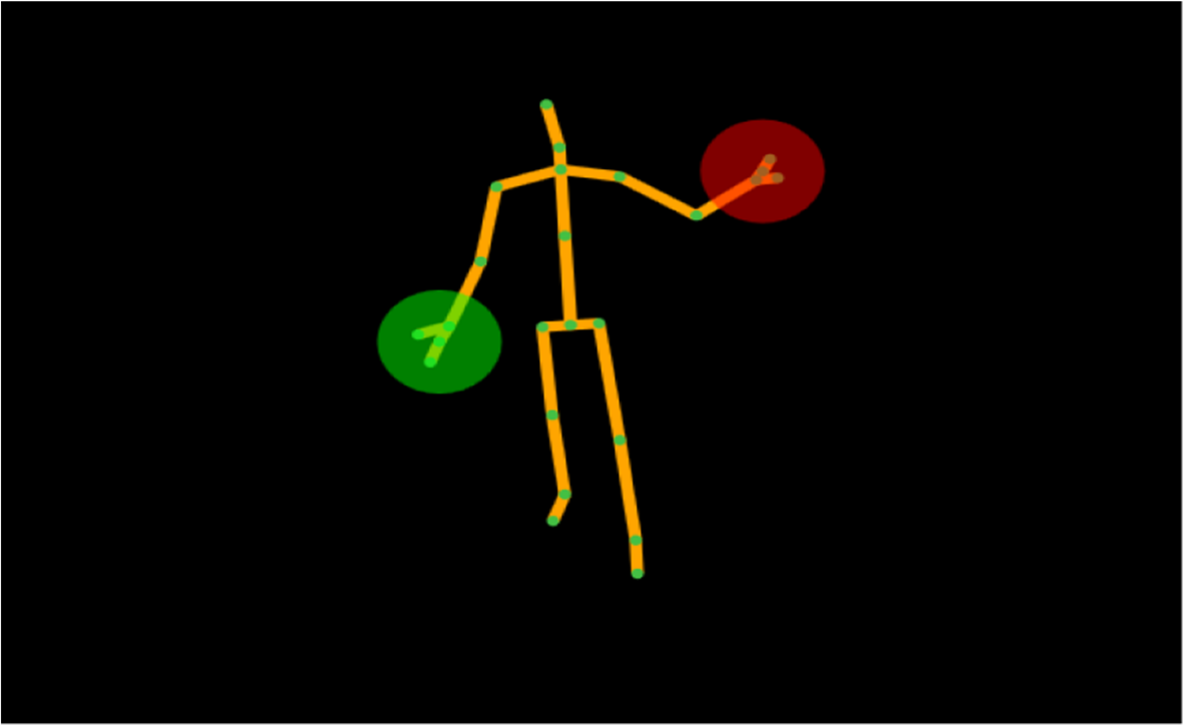
In our proposed setup, an automatic body representation is created based on which calculations for movement parameters can be automatically performed, for example step length, step width, joint angles and walking speed

We performed a qualitative study design using semi-structured interviews to explore the patient’s opinion on home-based video recording using vision-based movement analysis (Fig. 1) for medical purposes. We propose a set-up using a Kinect camera which objectively, continuously and non-obtrusively measures motor functioning (i.e. step length, step width, joint angles, walking speed etc.). This system automatically extracts these motor functioning parameters and is suitable for long-term monitoring (including e.g. during sleep) of free movements in the home situation. The system is based on a 3D depth camera (Kinect 2nd generation), with extension and optimization of the Kinect skeleton detection algorithms in order to enable the assessment of movement parameters including standing up and several gait parameters. These algorithms are currently under development.

The study was conducted at the Radboud University Medical Centre Nijmegen, the Netherlands. Parkinson’s patients, who previously (but not currently) participated in a research project in our centre and expressed willingness to participate in future research were invited via e-mail to partake.

All interviews were conducted in Dutch, taking approximately 40 min. The interviews were semi structured and included a standardized introduction, open-ended questions and prompts to encourage further discussion and more specific answers. Patients were informed about the proposed set-up with the Kinect camera (which, at the time of the interviews, was in developmental stage). Automated data extraction was discussed as a potential future option. However, we explicitly asked patients to give their opinion on collecting videos without this option of automated data extraction. Interviews were conducted by NMV between November 2015 and February 2016.

### Analysis

The interviews were recorded by a voice recorder and then transcribed verbatim by KS and JH. The qualitative data was analysed using Atlas.Ti v7 software (Scientific Software Development GmbH, Berlin, Germany). We implemented a framework method with deductive and inductive forms to analyse transcripts of the semi structured interviews [13]. This means that we both analyzed the data using the themes of the questionnaire as a starting point (deductive analysis) as well as an open approach in which we coded the data without any assumptions (inductive analysis). A bottom-up approach was used to create first-level themes and second-level codes with open coding. Categories were created by axial coding based on discussion with raters to establish consensus on categorizations [14]. A second rater independently assessed all recordings and any disagreements on themes was discussed until agreement was reached. Interviews were performed and analyzed until saturation was reached and no new concepts emerged from new interviews. We considered reaching saturation as a minimum number of three interviews not resulting in new information [15].

## Results

Saturation was reached after interviews with 16 PD patients (8 women) with a mean age of 61 years, and an average disease duration of 8.5 years.

All interviewed patients agreed that video recording at home is acceptable when a number of requirements are fulfilled. Three first-level themes were extracted (Table 1). These three themes (i.e. Camera recording, privacy protection and perceived motivation) will be discussed in more detail below.

**Table 1:** Overview of the results of the interviews categorized into themes, categories and a summary

Themes	Categories	Summary
Camera recording	Control over the camera	Patients indicate that they would like to control when the camera is turned on and off (81%). It would not be a problem if the camera is visible (69%) and patients would consent for continuous measurements of 2 weeks up to 3 months (100%).
	Visibility of the camera	
	Recording duration	
Privacy protection	Patient’s behaviour	Patient’s behaviour would probably be affected by awareness of the camera in the beginning (62%), but this will quickly reduce to normal. It is important that not only patients consent to being filmed, but also partners and depending on the location other people visiting the house. The living room was acceptable for all patients (100%).
	Partner’s consent	
	Camera location	
Perceived motivation	Contribution in science	The motivation of patients to participate in these projects relate to contributing to science and thereby helping future patients (100%). A potential personal benefit, when these video techniques would be used to give feedback on functioning to their treating physician, would also be a strong motivation.
	Helping other patients	
	Personal benefits	

### Camera recording

The large majority of patients (81%) indicated that it would be important to have a sense of control over the camera. For example, by having the possibility to turn the camera on and off themselves.

*“Actually, it’s crucial to be able to turn the camera on and off whenever I feel like having a private moment.”* (patient 16)

In addition, 50% of patients said they would like to see the recordings (and maybe even delete footage) before it would be shared with researchers or medical personnel. On the other hand, the other eight patients (50%) were concerned that interfering with the recordings would bias the results and therefore did not think this function would be necessary.

*“It should be possible to watch the record first by yourself and possibly delete parts.”* (patient 11)

*“There is no need to delete recordings. I would like to be filmed as I am. You won’t make it public.”* (patient 4)

Concerns regarding the camera location and visibility were minor; five patients (31%) suggested that the camera should not be visible to avoid influencing their natural behaviour. One patient suggested that the camera should not capture any sounds to increase privacy.

*“No, I don’t need to be informed if the camera is switched on or off, because maybe it would affect my behaviour.”* (patient 1)

Two weeks of continuous recording was found to be acceptable by all interviewed patients. All patients indicated that prolonging this period was also possible. However, three months of continuous recording was recognized as the upper limit.

*“I would have trouble to accept three months of constant filming.”* (patient 6)

### Privacy protection

10 participants (62%) said their behaviour would probably be temporarily influenced by having a camera in their home. They did perceive their behaviour to return to normal after getting used to being recorded. Five patients (31%) indicated that they expected their behaviour not to be influenced by the video recording.

*“I think it’s a matter of habituation. In a certain time, I will simply forget about the camera.”* (patient 5)

When asked about recording in different places of the house, interestingly, all patients agreed that installing a camera either in the living room or even bedroom would be acceptable. Seven (43%) patients suggested the kitchen would be the best place because they spend most of their time there. One patient indicated that the bedroom might be a very good location because it reduces the risk of unnecessary recordings of other people (who might be present in e.g. the living room). One other patient mentioned that more camera’s at the same time would be an intrusion of his privacy.

*“The camera should be placed at the place where I spend a lot of time, in the kitchen for example, so it will record everything that you want to know.”* (patient 6)

*“I don’t want a camera in every room at the same time, I need to have some privacy somewhere.”* (patient 8)

Patients also mentioned that the opinion and/or consent of their partners should be taken into consideration as they will be recorded as well. Similarly, visitors should also be addressed; they should be either asked for their consent or it should be possible to turn off the camera when guests arrive.

*“Other people should be kept out of the video as much as possible... but this is probably not possible, so my partner needs to consent as well”* (patient 7)

*“If you are sleeping in the same room, you are both being filmed. That’s why it would be important for me to ask for my partner’s opinion.”* (patient 12)

### Perceived motivation

All patients were willing to allow a camera in their homes for continuous monitoring. The main motivation for this was the common belief that only improving knowledge about the disease can, in the end, lead to a cure. Contributing to research was therefore seen as very important to all interviewed patients.

*“I would agree to be video recorded because I believe the observations could help future medicine.” (patient 7)*

Also, potentially improving personal treatment and helping future patients was a strong motivation to participate.

*“I would love to participate if it would benefit my own treatment. When you visit a neurologist you really need to describe the problem the right way and you never know exactly how often symptoms appear.” (patient 10)*

## Discussion

Here, we found that continuous video recording in the home situation may be an acceptable tool to gain insight in day to day functioning of patients with Parkinson’s disease. While we expected concerns about privacy using this technique, all interviewed patients were surprisingly positive about at home video monitoring. This is an important motivator to further develop, test and implement advanced home recording systems that can advance medical science. However, patients did point to several specific conditions that need to be taken into account when implementing video techniques. Important examples include giving patients control over the camera and the recording and protecting the privacy of family life. Both improvement of personal medical treatment and contribution to science were strong motivators to allow a certain extent of privacy violation. Having the camera record only for specific times during the day could potentially help to limit the privacy concerns and the need to turn off the camera. The total duration of the recordings should be sufficient to collect reliable and meaningful data. Here, patients were willing to accept to be video-recorded for at least two weeks. This time period is quite reasonable to gain insight into daily functioning, including fluctuations within the day and any day-to-day variability. Camera location will depend on the type of data that needs to be collected to answer a specific research or clinical question. For example, if the focus is on sleep, then preferably, the camera should be installed in the bedroom. The different rooms in a home all have their specific advantages and disadvantages. In the kitchen, for example, a lot of time is spent, so more information on fluctuations in functioning (even within a day) can be gathered. On the other hand, people other than the patient may also be present in the kitchen on various occasions. They either need to provide consent to be filmed or the camera should be turned off, reducing the amount of video material. The bedroom, on the other hand, will probably not involve many other people, but much less time is spent there and the time spent may be dedicated to activities that patients do not want to have recorded, e.g. getting dressed. Nonetheless, patients indicated to be open to different locations in their homes.

Despite the overall positive appraisal of home-based video monitoring by the patients participating in this study, we can not generalize these findings to all patients with PD. First, selection bias may have played a role, because we selected patients that were known to be interested is research. Second, the opinion of PD patients in the Netherlands (where care for patients with PD is generally well organized and innovative) may not necessarily reflect the opinion of patients located in another country. Finally, we did not have access to specific medical details on, for example, disease severity of the participating patients. Therefore we do not know whether these results apply to patients with PD in different disease stages.

Although the patients in our interviews did not mention data storage and access to the recordings as a potential barrier for their willingness to be filmed, it is obvious that this needs careful consideration and attention. Patients also implicitly assumed that this is taken care of. One of the patients indicated that the recordings would not be made public, meaning that the videos should only be accessible to certified persons with a clear access goal. Giving the patient a proper explanation considering this goal and the way in which the data are processed is therefore very important. There are various ways to solve this issue. For example, automatic vision-based movement analysis does not require to store the real patient videos. Automatic analyses can be performed in real-time based on the body representation and joint positions only (Fig. 1) [16]. At this point in the development process, the full videos may still be needed to interpret e.g. unusual findings, but once development and validation has been done, only extracted parameters on movement patterns will need to be shared.

## Conclusion

Home- based video monitoring for medical or scientific purposes may be acceptable for patients with PD. The opinions presented here were all positive. However, this does not mean that we can extrapolate these findings to the general population of patients with PD. We included patients who are interested in research and may therefore be positively biased. Yet, the present results do indicate that home-based video measurements do not need to be ruled out in advance because of ethical and privacy considerations.

## Abbreviations

PD: Parkinson’s disease
UPDRS: Unified Parkinson’s Disease Rating Scale
